# Clinical outcomes following concomitant arthroscopic rotator cuff repair and anterior shoulder stabilization: a systematic review and meta-analysis

**DOI:** 10.1016/j.xrrt.2026.100868

**Published:** 2026-08-28

**Authors:** Dhruv Limaye, Andy Contreras, Tim Liu, Thomas J. Kremen

**Affiliations:** Department of Orthopaedic Surgery, University of California - Los Angeles, Los Angeles, CA, USA

**Keywords:** Shoulder instability, Rotator cuff repair, Bankart repair, Arthroscopy, Concomitant repair, Systematic review, Meta-analysis

## Abstract

**Background:**

To evaluate clinical outcomes, including patient-reported function, recurrent instability, and complication rates, following concomitant arthroscopic rotator cuff repair and anterior shoulder stabilization.

**Methods:**

A systematic review was performed in accordance with Preferred Reporting Items for Systematic Reviews and Meta-Analyses 2020 guidelines. PubMed, Embase, and Cochrane CENTRAL were searched from inception through April 2026. Studies evaluating concomitant arthroscopic rotator cuff repair and anterior stabilization with reported post-operative outcomes were included. Pooled proportions for recurrent instability, structural rotator cuff retear, and reoperation were calculated using random-effects meta-analysis with logit transformation and Knapp–Hartung adjustment.

**Results:**

Six studies (160 patients) met inclusion criteria (Level III–IV). The pooled recurrence rate was 3.3% (95% confidence interval [CI], 1.0%-9.9%; I^2^ = 0%). The pooled rotator cuff retear rate was 12.0% (95% CI, 3.4%-35.0%; I^2^ = 4.1%). The pooled reoperation rate was 5.6% (95% CI, 1.2%-22.2%; I^2^ = 0%). Post-operative functional scores exceeded established thresholds in cohorts with predominantly acute instability, while the sole chronic-predominant cohort fell below the Constant-Murley success threshold.

**Conclusion:**

Concomitant arthroscopic rotator cuff repair and anterior shoulder stabilization is associated with low rates of recurrent instability but a meaningful risk of structural rotator cuff failure. Outcomes appear to be influenced by instability chronicity, with acute presentations demonstrating more favorable results. Prospective studies stratified by instability chronicity and with standardized imaging surveillance are needed.

Anterior shoulder instability is a common orthopedic condition that can lead to recurrent dislocation, functional impairment, and long-term degenerative changes if left untreated.^19^^,^^46^^,^^47^ Arthroscopic anterior stabilization, most commonly through Bankart repair, has shown low recurrence rates with reliable clinical outcomes in appropriately selected patients.^22^^,^^29^^,^^30^ However, in older or higher-energy injury populations, the clinical presentation and treatment considerations differ as anterior dislocation is frequently associated with concomitant rotator cuff pathology, in this population.^14^^,^^39^ Rotator cuff tear rates following anterior shoulder dislocation have been reported in 35% to 86% of patients over 40 years of age, in contrast to the younger athletic population who predominate in isolated instability literature.^14^^,^^23^^,^^39^

Prior studies have shown that concomitant rotator cuff repair is required in a substantial proportion of older patients undergoing surgical management for anterior instability, demonstrating the clinical relevance of this combined pathology.^28^ In this population, shoulder dysfunction is often driven by disruption of both static stabilizers (capsulolabral complex) and dynamic stabilizers (rotator cuff musculature). Failure to address associated rotator cuff pathology can result in persistent pain, weakness, and suboptimal functional recovery, even after successful stabilization.^14^ In contrast, isolated rotator cuff repair may not adequately restore stability in the presence of capsulolabral injury. Thus, combined lesions represent a distinct clinical scenario requiring restoration of both static and dynamic components of shoulder stability.

Surgical management of combined rotator cuff tear and anterior instability is under characterized. Single-stage arthroscopic repair has been increasingly reported, with multiple small case series demonstrating favorable outcomes.^22^ However, combined procedures introduce competing biomechanical and rehabilitation demands, as rotator cuff repair improves with early controlled motion to minimize stiffness, while anterior stabilization requires protected immobilization to promote capsulolabral healing.^34^^,^^35^ Furthermore, the existing literature is limited by small sample sizes; heterogeneous patient populations; variability in instability chronicity; and inconsistent reporting of complications such as post-operative stiffness, structural cuff failure, and reoperation.^22^^,^^34^ However, no prior study has provided pooled estimates of key clinical outcomes, including recurrent instability, structural rotator cuff retear, and reoperation rates in this population and evidence-based recommendations are poorly defined.

To our knowledge, no systematic review or meta-analysis has comprehensively evaluated outcomes following concomitant arthroscopic rotator cuff repair and anterior shoulder stabilization to date. Given the relative rarity of this combined pathology and the limited power of individual studies, pooled analysis may provide more reliable estimates of clinical outcomes and complications. Therefore, the purpose of this study was to perform a systematic review and proportional meta-analysis to evaluate patient-reported outcomes, recurrent instability, and complication rates following concurrent rotator cuff repair and anterior shoulder stabilization. We hypothesized that combined surgical management would result in durable anterior stabilization with recurrent instability rates comparable to isolated Bankart repair, while structural rotator cuff retear would emerge as the primary structural complication.

## Materials and methods

### Search strategy and study selection

The Preferred Reporting Items for Systematic Reviews and Meta-Analyses guidelines were followed for this study.^25^ This review was not prospectively registered on the International Prospective Register of Systematic Reviews (PROSPERO); however, the study protocol and eligibility criteria were defined prior to data extraction and the review was initiated before registration was feasible. On February 20, 2026, a systematic search was performed across the electronic databases of PubMed, Embase, and Cochrane CENTRAL. The comprehensive search algorithms and database-specific syntax used for this review are detailed in Appendix 1.

The search was limited to studies published in English due to feasibility constraints related to translation and data extraction. Duplicate studies (n = 337) were removed. On April 1, 2026, the literature search was repeated to ensure that no new articles had been published in the literature. Two reviewers independently manually screened the titles and abstracts of all studies for inclusion and then reviewed the full texts according to the inclusion and exclusion criteria using the screening software Rayyan, with discrepancies resolved by consensus.^24^ Agreement for inclusion and exclusion was reached for all studies, and a third independent reviewer was not necessary.

Inclusion criteria consisted of the following: (1) studies reporting clinical outcomes following concomitant arthroscopic repair of both a rotator cuff tear and anterior shoulder instability (eg, Bankart lesion), (2) structural pathology including acute full-thickness rotator cuff tears (studies containing high-grade partial-thickness tears involving >50% of the footprint were included only if they were surgically completed and formally repaired as full-thickness defects), and (3) reporting of post-operative patient-reported outcome measures (PROMs) or sufficient data to calculate functional success proportions. Studies were eligible regardless of whether the initial surgical indication was for an acute, primary dislocation or for chronic, recurrent instability. Exclusion criteria consisted of animal or cadaveric studies, isolated rotator cuff repairs, isolated Bankart repairs, open surgical procedures, biomechanical studies, and systematic reviews.

### Data extraction and quality assessment

Two independent reviewers performed data extraction into a standardized, predesigned spreadsheet, with any discrepancies resolved through consensus or consultation with a third senior reviewer. Extracted variables were categorized into 3 primary domains to ensure a comprehensive analysis of the concomitant procedures.

#### Study and patient characteristics

Study metadata and patient demographics included primary identifiers (author, year, country), study design, and the Level of Evidence (I–IV), and isolated sample size (n) of the concomitant Bankart and rotator cuff repair group. The patient profile consisted of age (mean/median), gender distribution (% male), body mass index, and the chronicity of pre-operative instability (proportion of patients with acute primary dislocations).

#### Surgical and lesion morphology

Surgical characteristics extracted included the specific rotator cuff tendons involved (supraspinatus, infraspinatus, subscapularis), tear size or retraction, the number of anchors utilized for both the Bankart and rotator cuff repair components, anchor material, and any concomitant long head of the biceps tendon procedures.

#### Clinical outcomes and safety

Clinical outcomes focused on follow-up duration (mean), pre-operative and post-operative PROMs, and post-operative range of motion (ROM) deficits compared to the uninjured contralateral shoulder. Safety and durability metrics included the rates of recurrent anterior instability, clinical post-operative stiffness requiring intervention (eg, manipulation under anesthesia or capsular release), and structural rotator cuff retear rates.

For studies that reported mean continuous functional scores but failed to report a standard deviation, variance imputation was utilized to allow for inclusion in the quantitative analysis.^12^^,^^18^ In accordance with the guidelines set forth in the *Cochrane Handbook for Systematic Reviews of Interventions*, missing standard deviations were imputed by borrowing estimates from the most methodologically similar study reporting the same outcome.^7^

The methodological quality of the included studies was assessed using the Methodological Index for Non-Randomized Studies.^38^ For non-comparative studies, a maximum score of 16 was utilized to evaluate the risk of bias. We considered non-comparative studies with scores >12 to be high quality, and 8-12 to be moderate quality.

### Outcome hierarchy for primary analysis

Hierarchy prioritized the American Shoulder and Elbow Surgeons (ASES) score, followed by the Constant Murley score (CMS),^31^ the Rowe score,^32^ and finally the University of California, Los Angeles (UCLA) Shoulder Rating Scale. Isolated, single-study metrics (eg, Single Assessment Numeric Evaluation, Single Assessment Numeric Evaluation, University of Pennsylvania Shoulder Score) were excluded from pooling to maintain statistical integrity.

### Statistical analysis and meta-analysis

All statistical analyses were performed using R (version 4.5.2; R Foundation for Statistical Computing, Vienna, Austria). Data management and initial calculations were performed in Microsoft Excel (version 2409; Microsoft Corporation, Redmond, WA, USA). Meta-analyses were conducted using the metafor package (version 4.6-0) in R.^42^

Pooled proportions were estimated using a random-effects meta-analysis with the restricted maximum likelihood estimator for between-study variance (τ^2^).^21^ A random-effects model was selected a priori due to expected clinical heterogeneity among the included studies, including differences in patient demographics, chronicity of instability, and surgical technique.^7^ Proportions were modeled using a logit transformation to stabilize variances and ensure appropriate weighting of studies with varying sample sizes.^2^^,^^36^ Pooled estimates were subsequently back-transformed to the original proportion scale for interpretation to account for the small number of included studies, confidence intervals (CIs) for pooled estimates were calculated using the Knapp–Hartung adjustment, which provides more conservative variance estimation in random-effects meta-analysis. If a study cohort included patients who did not undergo the strict concomitant repair, and the clinical outcome data for the combined procedure subgroup could not be mathematically isolated, the study was included for qualitative narrative synthesis but explicitly excluded from the quantitative proportional meta-analysis.^17^

### Heterogeneity and bias

Statistical heterogeneity was assessed using the I^2^ statistic, with I^2^ > 50% indicating significant heterogeneity and I^2^ > 75% representing high heterogeneity. Publication bias and small-study effects were assessed via visual inspection of funnel plots to evaluate symmetry.^11^ Funnel plot asymmetry was assessed quantitatively using Egger regression test within a mixed-effects meta-regression framework.^11^ Trim and fill analysis was conducted as a precautionary measure using a random-effects model to evaluate the potential impact of unpublished studies on the pooled proportional success rates, regardless of Egger test results.^10^

## Results

### Study selection and patient demographics

The Preferred Reporting Items for Systematic Reviews and Meta-Analyses flowchart (Fig. 1) shows the inclusion and exclusion criteria applied to the 1,162 published articles that were identified in the initial search. After removal of duplicates, 825 records were screened, resulting in 35 full-text articles assessed for eligibility. In total, 6 studies met the final inclusion criteria for the systematic review, representing a pooled cohort of 160 unique patients.

**Figure 1:**
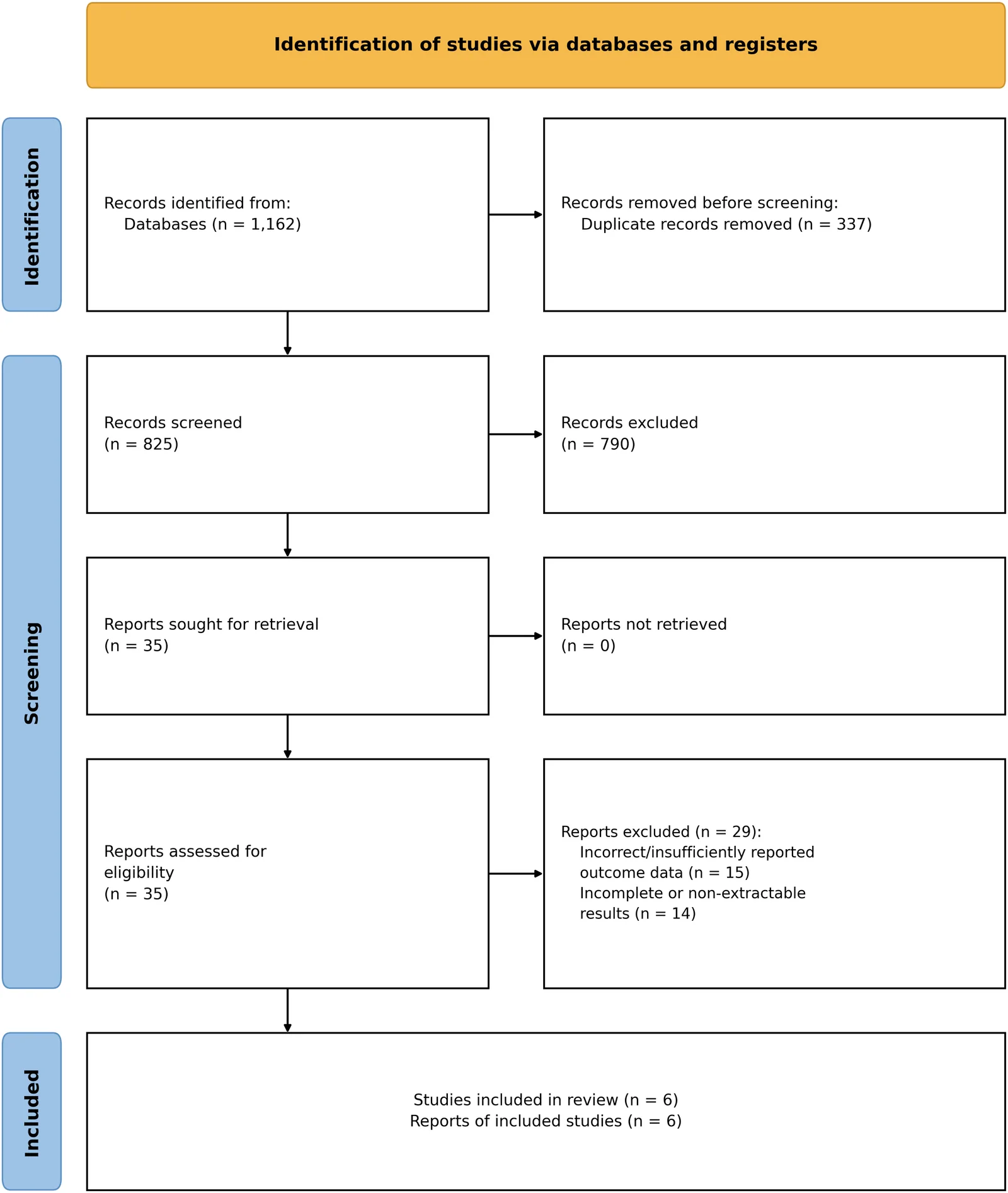
PRISMA flow diagram. The PRISMA flow diagram represents the search and selection procedure applied during the literature review for the identification of studies reporting on concomitant arthroscopic rotator cuff repair and anterior shoulder stabilization. *PRISMA*, Preferred Reporting Items for Systematic Reviews and Meta-Analyses.

The pooled study population was predominantly male (66.8%) with a weighted mean age of 53.1 years (range: 47.0-58.8 years and mean clinical follow-up averaged 48.2 months across the analyzed cohort (range: 27.2-64.8 months). Pre-operative instability chronicity was reported for a subset of 127 patients. Of these patients, 48% presented following an acute primary dislocation, while 52% presented with chronic recurrent instability (>2 episodes) (Fig. 2). Supraspinatus involvement was the predominant tendon injured across reporting cohorts, though substantial infraspinatus and subscapularis involvement was also observed, particularly in studies with higher proportions of chronic instability (Fig. 3).

**Figure 2:**
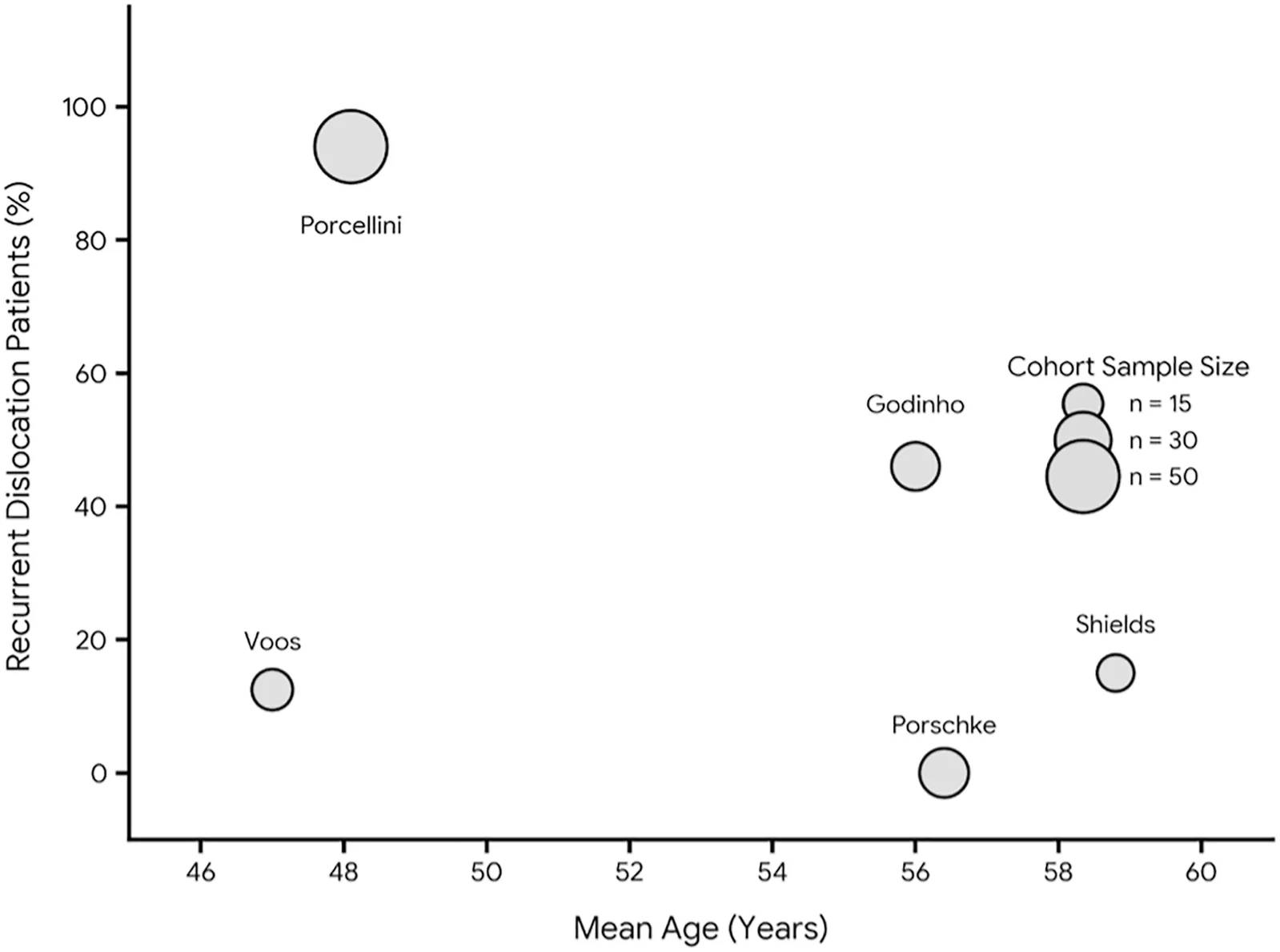
Instability chronicity vs. mean age by study cohort. Bubble plot demonstrating the relationship between the mean age of the included study cohort and the proportion of patients presenting with chronic, recurrent instability. The size of each bubble is proportional to the cohort sample size (n). An apparent inverse trend is observed between mean cohort age and chronicity proportion, consistent with 2 distinct clinical phenotypes: younger patients with chronic/attritional instability versus older patients presenting after acute high-energy dislocations.

**Figure 3:**
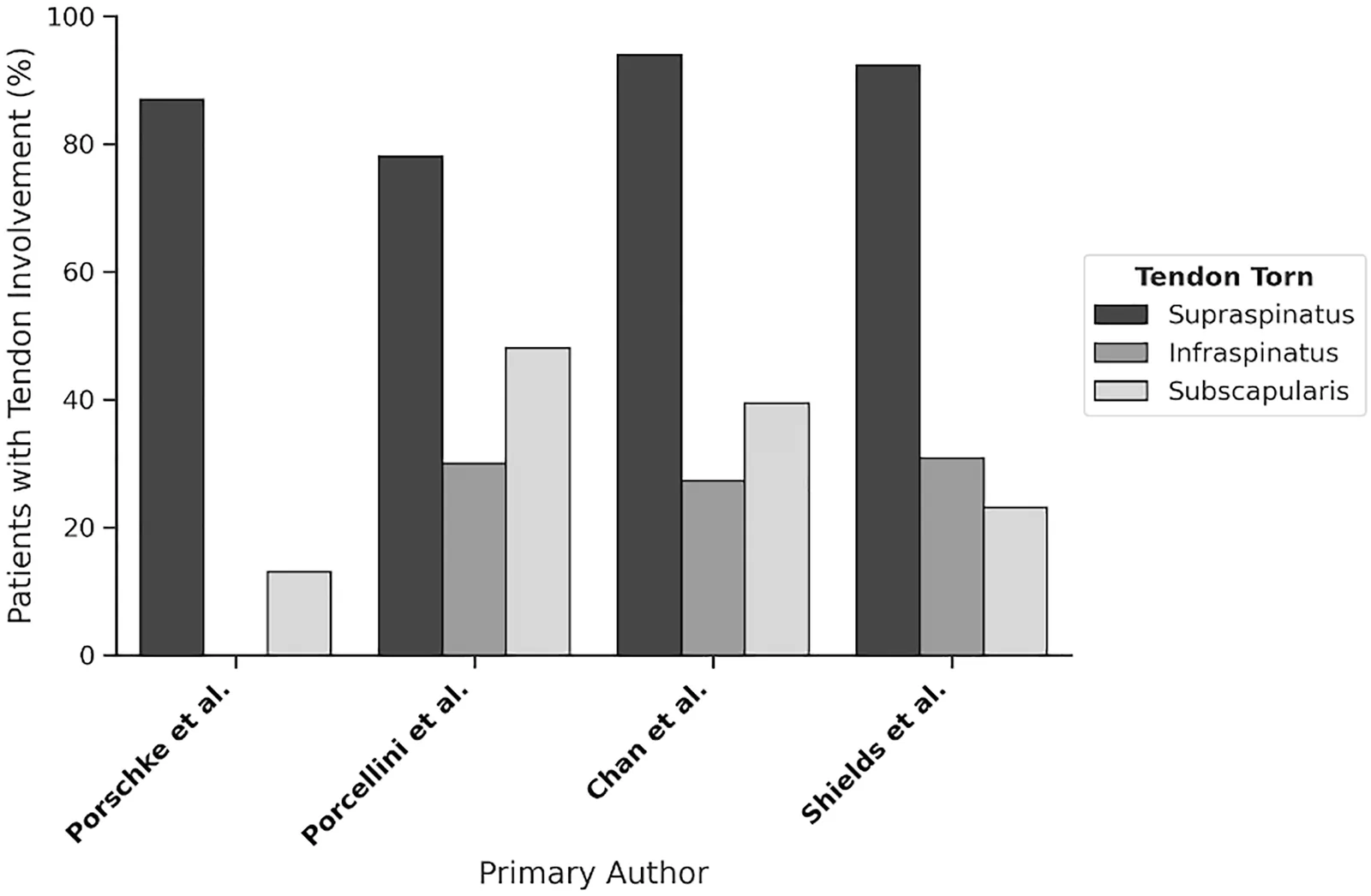
Distribution of specific rotator cuff tendon involvement across included cohorts. Horizontal bar chart demonstrating the proportion of specific rotator cuff tendons torn within each study's combined repair cohort. The supraspinatus was consistently the most frequently involved tendon across all reporting studies, reflecting its universal vulnerability in these concomitant injury patterns. Rates of subscapularis and infraspinatus involvement demonstrated higher variability, highlighting the clinical heterogeneity of the tear morphologies. (Note: Godinho et al and Voos et al are not depicted as they did not report the specific anatomical breakdown of tendon involvement for their combined cohorts).

### Patient-reported outcomes and global function

Given the substantial heterogeneity in PROM scores reported across the studies, including the ASES score, the CMS, the Rowe score, and the UCLA shoulder score, post-operative functional outcomes are presented as a narrative synthesis with data reported using normalized scores (Table I). Quantitative meta-analysis was performed for complications (recurrent instability, structural retear, and all-cause reoperation), for which consistent data were available across all included studies.

**Table I:** Narrative synthesis table.

Study (yr)	n	Mean age (yr)	Instability chronicity (acute %)	Chronicity category	Surgical technique	PROM instrument (Hierarchy)	Post-op score (mean ± SD)	Scale range	Success threshold	Threshold met?	Dominant Tendon Involvement	Follow-up (mo)	MINORS score
Chan et al (2020)^6^	36	56.8	NR	Mixed (mean 1.7 dislocations)	Arthroscopic Bankart repair (subset with bony Bankart/glenoid augmentation) + RCR	ASES score	83.4 ± 22.5	0-100	≥80 (success)	Yes	Supraspinatus 94% Infraspinatus 27% Subscapularis 39%	24	9/16
Godinho et al (2016)^13^	22	56	54%	Mixed (54% first-time)	Arthroscopic Bankart repair + RCR	UCLA shoulder score	33.96 ± 1.77	0-35	≥28 (good)	Yes	NR	39	10/16
Porcellini et al (2006)^26^	50	48.1	6%	Chronic (94% recurrent)	Arthroscopic Bankart + RCR	Constant Murley score (CMS)	69.4 ± 11.1	0-100	≥80 (good)	No	Supraspinatus 78% Infraspinatus 48% Subscapularis 48%	36	10/16
Porschke et al (2020)^27^	23	56.4	100%	Acute (100% first-time)	Arthroscopic Bankart repair (8 converted to mini-open) + RCR	Constant Murley score (CMS)	85.1 ± 14.7	0-100	≥80 (good)	Yes	Supraspinatus 87% Subscapularis 13%	58	13/16
Shields et al (2014)^37^	13	58.8	85%	Acute (85% first-time)	Arthroscopic Bankart repair + RCR	Constant Murley score (CMS)	80.5 ± 18.9	0-100	≥80 (good)	Yes	Supraspinatus 92% Infraspinatus 31% Subscapularis 23%	39	13/16
Voos et al (2007)^44^	16	47	75%	Mixed (∼75% first-time)	Arthroscopic Bankart repair + RCR	ASES score	95.8 ± 22.5	0-100	≥80 (success)	Yes	Supraspinatus dominant	32	10/16

*PROM*, patient-reported outcome measure; *SD*, standard deviation; *MINORS*, Methodological Index for Non-Randomized Studies; *ASES*, American Shoulder and Elbow Surgeons.; *RCR*, rotator cuff repair; *UCLA*, the University of California, Los Angeles; *NR*, not reported.

Among the 6 included studies, Godinho et al^13^ reported a mean post-operative UCLA score of 33.96 ± 1.77 (threshold ≥28; normalized score 85.0%), reflecting consistently excellent functional outcomes in their cohort of 22 patients. Porschke et al reported a mean CMS of 85.1 ± 14.7 and a mean Rowe score of 82.4 ± 15.2 in their exclusively acute cohort of 23 patients, with both scores exceeding established success thresholds (CMS ≥80; Rowe ≥75). Shields et al^37^ reported a mean ASES of 89.7 ± 12.6 and a mean CMS of 80.5 ± 18.9 in their cohort of 13 patients that included predominantly acute dislocations (85%).Voos et al reported a mean ASES of 95.8 ± 22.5 in their cohort of 16 patients. Chan et al^6^ reported a mean post-operative ASES of 83.4 ± 22.5 across 36 patients with a mean of 1.7 prior dislocations, meeting the established success threshold. In contrast, Porcellini et al^26^ reported a cohort was composed of 94% patients with chronic recurrent instability, with a mean of 2-10 prior dislocations and reported a mean CMS of 69.4 ± 11.1 and a mean Rowe score of 78.9 ± 15.2. Notably, the mean CMS fell below the established good-outcome threshold of 80, and was the only study cohort in this review to do so.

Across all 6 studies, a trend was observed between the chronicity of instability and functional outcome. The 3 study cohorts composed of predominantly acute or primary-dislocation patients (Porschke, Shields, Voos), each reported mean PROMs above success thresholds, while the sole cohort dominated by chronic recurrent instability (Porcellini) fell below the CMS threshold.

### Recurrent instability

Recurrent anterior instability was explicitly captured across all 160 patients. Across 6 studies (N = 160), 3 cases of recurrent instability were reported, corresponding to study-level rates ranging from 0% to 4.3% (Table II). Pooled meta-analysis of all 6 studies (N = 160) using a random-effects model with restricted maximum likelihood estimation and logit transformation yielded a model-based pooled proportion of recurrent anterior instability of 3.3% (95% CI: 1.0%-9.9%), with homogeneous between-study variance (I^2^ = 0.0%) (Fig. 4*A*). No significant funnel plot asymmetry was detected on Egger regression test (*P* = .692), and trim-and-fill analysis estimated zero missing studies, with an unchanged adjusted estimate of 3.3% (95% CI: 1.4%-7.7%).

**Table II:** Summary of pooled estimates for meta-analyzed outcomes.

Outcome	k (studies)	N (patients)	Events (n)	Pooled estimate (95% CI)	I^2^	Egger p
Recurrent instability	6	160	3	3.3% (1.0%-9.9%)	0.0%	0.692
Structural rotator cuff retear	5	124	12	12.0% (3.4%-35.0%)	4.1%	0.100
All-cause reoperation	4	131	6	5.6% (1.2%-22.2%)	0.0%	0.215

*CI*, confidence interval.

**Figure 4:**
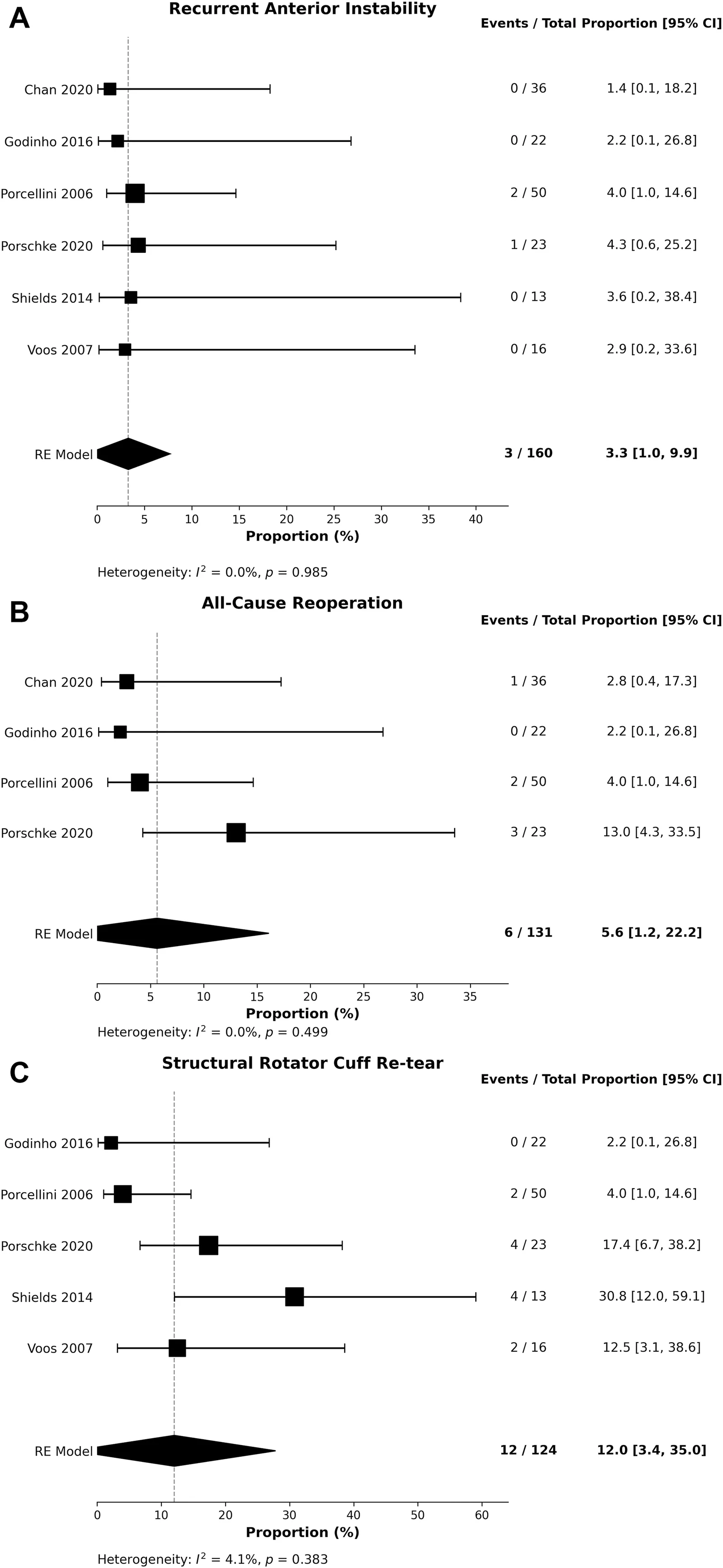
Proportional meta-analyses of post-operative complications and failure modes. Composite forest plots displaying the pooled incidence of adverse clinical outcomes following single-stage combined arthroscopic rotator cuff and anterior labral repair. (**A**) Recurrent anterior shoulder instability: model-based pooled estimate 3.3% (95% CI: 1.0%-9.9%; I^2^ = 0.0%; k = 6, N = 160; 3 observed events). (**B**) Structural rotator cuff retear: model-based pooled estimate 12.0% (95% CI: 3.4%-35.0%; I^2^ = 4.1%; k = 5, N = 124; 12 observed events). (**C**) All-cause reoperation: model-based pooled estimate 5.6% (95% CI: 1.2%-22.2%; I^2^ = 0.0%; k = 4, N = 131; 6 observed events). Each square represents the point estimate of an individual study, with the size of the square indicating the relative weight of the study in the random-effects meta-analysis. Horizontal lines denote 95% confidence intervals. The diamond at the *Bottom* of each plot represents the overall pooled proportion. The data demonstrates that structural failure of the rotator cuff tendon (**B**) is the dominant failure mode, occurring at a notably higher rate than recurrent capsulolabral instability (**A**). (Note: Studies that did not report the specific complication of interest were excluded from that respective subgroup's plot). *CI*, confidence interval.

Of the 3 recurrence events, 1 occurred in the Porschke cohort (1/23, 4.3%) and 2 in the Porcellini cohort (2/50, 4%). In both recurrent instability cases reported by Porcellini et al,^26^ revision surgery via the open Bristow-Latarjet procedure was performed, at which point irreparable rotator cuff tears were identified in both patients, underscoring the potential for compounded failure in this population.

### Structural rotator cuff retear

Structural rotator cuff retear or failure was reported in 4 of 6 studies, as Chan et al did not report retear outcomes and was excluded from this analysis (k = 5, N = 124) (Table II). Pooled meta-analysis yielded a model-based estimate of rotator cuff failure of 12.0% (95% CI: 3.4%-35.0%), with low between-study heterogeneity (I^2^ = 4.1%) (Fig. 4*B*).

Retear rates varied substantially across the studies and likely reflects a relationship with the method of post-operative surveillance. Shields et al^37^ employed routine post-operative ultrasound evaluation in all patients and identified persistent or recurrent full-thickness cuff defects in 4 of 13 patients (30.8%).In contrast, Porcellini et al identified retears at the time of revision surgery for recurrent instability and reported a retear rate of 4.0% (2/50). Porschke et al^27^ reported retears in 4 of 23 patients (17.4%) and Voos et al^44^ in 2 of 16 patients (12.5%), while Godinho et al^13^ reported no structural failures (0/22). Leave-one-out sensitivity analysis demonstrated that omission of Shields, which had the highest retear rate and the most systematic imaging surveillance, reduced the pooled estimate to 8.9% (95% CI: 2.1%-31.2%), while omission of Godinho increased it to 14.1% (95% CI: 3.3%-43.7%). This confirmed that the wide CI on the pooled estimate reflects genuine surveillance-driven heterogeneity rather than a single outlier.

### All-cause reoperation

All-cause reoperation was reported in 4 of 6 studies. Shields et al^37^ and Voos et al^44^ did not report reoperation outcomes and were excluded from this analysis (k = 4, N = 131) (Table II). Pooled meta-analysis yielded a model-based estimate of reoperation rate of 5.6% (95% CI: 1.2%-22.2%), with low between-study heterogeneity (I^2^ = 0.0%) (Fig. 4*C*). Porschke et al^27^ reported the highest individual reoperation rate (3/23, 13.0%), of which 2 reoperations among 13 arthroscopic patients (15.4%) were driven by severe post-operative stiffness requiring arthroscopic capsular release, despite employing passive range-of-motion exercises from post-operative day 1; the third reoperation occurred in the mini-open subgroup. Porcellini et al reported reoperation in 2 of 50 patients (4.0%), both for recurrent anterior instability treated with open Bristow-Latarjet revision, and at reoperation, both patients were found to have progressed to irreparable rotator cuff tears. Chan et al^6^ reported a single reoperation (1/36, 2.8%) for revision to anatomic total shoulder arthroplasty, while Godinho et al^13^ reported no reoperations.

### Post-operative stiffness, range of motion, and neurologic morbidity

Post-operative stiffness requiring surgical intervention was reported in only 1 study, so formal meta-analysis was not conducted. Porschke et al^27^ reported clinically significant stiffness requiring arthroscopic revision in 2 of 13 arthroscopic patients (15.4%).ROM deficits were also reported by Porschke et al, who reported deficits in external rotation relative to the contralateral shoulder (42° vs. 53°), alongside deficits in forward flexion (157° vs. 164°) and abduction (152° vs. 160°). In contrast, Voos et al reported minimal residual motion deficits at final follow-up (forward flexion 173° vs. 178°, external rotation 78° vs. 82°), with 77% of patients returning to preinjury athletic activity. Porcellini et al noted slight residual stiffness in a subset of patients but did not report the proportion affected or quantify the deficit.

Neurologic morbidity was reported by 2 studies. Godinho et al^13^ noted axillary nerve injury in 2 patients (9.1%), both presenting in the context of a terrible triad injury pattern (anterior dislocation with combined labral and rotator cuff disruption). Voos et al^44^ reported persistent abduction weakness in 6 patients (37.5%) at final follow-up. This rate substantially exceeded the structural retear rate in that same cohort (12.5%) which suggests that the functional deficit in this population is not fully explained by repair failure and may reflect residual neurapraxia or incomplete muscular recovery.

### Heterogeneity and publication bias

Statistical heterogeneity across the 3 meta-analytic outcomes was low to absent for recurrent instability (I^2^ = 0.0%) and reoperation (I^2^ = 0.0%), and low for structural retear (I^2^ = 4.1%). Egger regression test did not identify statistically significant funnel plot asymmetry for any of the 3 outcomes (*P* = .692, *P* = .100, and *P* = .215, respectively), and trim-and-fill analysis estimated zero missing studies in all 3 cases, providing no statistical evidence of publication bias (Supplemental Fig. S1). Sensitivity analyses confirmed that no single study exerted disproportionate influence on any pooled estimate; however, the wide CIs observed, particularly for retear (3.4%-35.0%) and reoperation (1.2%-22.2%), reflect the limited precision inherent to a pooled cohort of 124 and 131 patients, respectively, across 5 and 4 studies.

## Discussion

The most significant finding of this systematic review is that single-stage combined arthroscopic rotator cuff repair and anterior shoulder stabilization appears to achieve durable anterior stability, with a pooled recurrent instability rate of 3.3% (95% CI: 1.0%-9.9%), in a population for whom the primary operative indication is restoration of cuff function rather than prevention of recurrent dislocation. Across 6 studies comprising 160 patients, structural rotator cuff retear emerged as the dominant complication at a pooled rate of 12.0% (95% CI: 3.4%-35.0%). From a clinical perspective, these findings suggest that combined repair demonstrates consistent stabilization across included cohorts, but that factors such as older patient age, traumatic injury mechanism, and instability chronicity, represent the primary determinant of both structural and functional success in cuff healing. Given the rarity of this combined pathology and the limited size of the available evidence base, these findings should be interpreted as hypothesis-generating and is intended to provide the best available pooled estimates to inform future prospective investigation.

These findings are biologically plausible given the established relationship between anterior shoulder instability and rotator cuff pathology.^14^ Anterior glenohumeral dislocation disrupts the static stabilizers in the shoulder, including the labrum, capsule, and glenohumeral ligaments, while simultaneously forcing eccentric load on the dynamic stabilizers, particularly the supraspinatus and subscapularis rotator cuff muscles.^41^ The subscapularis, as the primary anterior dynamic stabilizer of the glenohumeral joint, is progressively stressed with each dislocation event, consistent with Turkel et al's^41^ framework of force-dependent stabilizing mechanisms. In older patients, this frequently produces full-thickness rotator cuff tears during instability events, creating a combined lesion in which both static and dynamic stabilizers are compromised. Therefore, single-stage combined repair is justified as restoring capsulolabral integrity can normalize glenohumeral kinematics and reduce translational stress on the repaired cuff, while the cuff repair restores the force-coupling that Bankart repair alone cannot provide.^14^^,^^5^

The population operated on in this review is fundamentally different from established instability literature, which is dominated by young athletes. Recurrence risk after first anterior dislocation declines from 72 to 100% in patients under 20 years to approximately 27% in patients over 30 years.^19^^,^^46^ Thus, older patients dislocate less frequently but sustain more severe structural injury with each episode. Previous literature demonstrates that only 26% of patients over 50 years old ultimately require surgical intervention.^39^ However, among those who do, all patients undergoing concomitant labral repair also required simultaneous cuff repair.^40^ The primary operative indication is therefore not specifically prevention of uncommon recurrent dislocation, but rather restoration of shoulder function compromised by the cuff tear itself.^40^ This contextualizes the finding of a 3.3% recurrent instability rate, as these patients were not at a high baseline redislocation risk and also because the arthroscopic Bankart component is technically reliable. This pooled rate is numerically similar to reported rates following isolated arthroscopic Bankart repair (5-15%) and the Latarjet procedure (2-5%).^1^ Similarly, the structural retear rate of 12.0% is comparable to rates reported following isolated rotator cuff repair in age-matched populations.^20^ However, these comparisons are indirect, drawn from separate patient populations, surgical eras, and study designs, and cannot establish whether the addition of rotator cuff repair affects stabilization efficacy.

The inversion of the expected complication profile, with a pooled recurrent instability rate of 3.3% versus a pooled structural retear rate of 12.0% suggests that the Bankart component achieved reliable stabilization while cuff healing emerged as the primary biological failure mode. Literature shows that cuff retear risk holds an odds ratio of 1.05 per year of age, roughly doubling from 15% at age 50 years to over 30% at age 70 years.^20^ The weighted mean age of 53.1 years in these cohorts fits the age cohort corresponding with elevated biological risk. Furthermore, both of Porcellini's revision Latarjet cases also revealed irreparable rotator cuff tears at reoperation which shows that combined repair failure may leave patients worse than either isolated failure alone. Shields et al,^37^ employed routine post-operative ultrasound and identified retears in 30.8% of patients compared to 4.0% in Porcellini's cohort where structural failure was measured only at revision operation.^26^ This pattern mirrors the broader isolated rotator cuff repair literature, in which systematic post-operative imaging consistently identifies structural failure at rates substantially exceeding those detected in clinic, suggesting that the pooled retear estimate of 12.0% almost certainly underestimates the true structural failure burden in this population.^8^^,^^33^ Routine post-operative structural imaging at 6-12 months may warrant consideration for standard practice in this population.

The review also shows the role of instability chronicity as a determinant of functional outcomes. Porschke et al, whose cohort comprised exclusively acute single-episode dislocations, achieved a CMS of 85.1 and Rowe score of 82.4, both meeting the established range for successful operation.^27^ In contrast, Porcellini et al, reported a cohort of 94% chronic dislocators with up to 10 prior dislocations, achieved a CMS of 69.4, falling below the success threshold.^26^ This pattern held across all 6 studies. Chronic instability causes progressive glenoid bone loss, capsular attenuation, and rotator cuff fatty infiltration, and each of these factors are associated with worse outcomes in both isolated Bankart repair and isolated rotator cuff repair.^3^^,^^5^^,^^15^ In this review, glenoid bone loss represents a consequential unmeasured confounder, as computed tomography–based quantification was absent from all 6 included studies.^16^^,^^45^ Significantly, functional outcomes worsened incrementally with glenoid bone loss, with significant decline demonstrated beginning at 13.5% bone loss.^4^ Chronicity also corresponded to tendon involvement in these cohorts. For example, Porcellini's chronic cohort had 48% subscapularis involvement versus 13% in Porschke's acute cohort, which reflects the progressive attrition of the primary anterior dynamic stabilizer due to dislocations.^26^^,^^27^ Thus, these findings show that older patients with an acute primary dislocation and a supraspinatus-dominant tear and minimal bone loss represent the most favorable combined repair candidate. However, patients with chronic recurrent instability, subscapularis and multi tendon involvement, and progressive bone loss warrants consideration of alternative strategies such as staged repair or Latarjet combined with cuff repair, given the biological and mechanical challenges of all-arthroscopic combined repair in this group.

The combined procedure of cuff repair and anterior stabilization creates a fundamental rehabilitation conflict where Bankart repair demands protected immobilization to allow capsulolabral healing, while rotator cuff repair demands early controlled motion to prevent stiffness. Shields et al reported a stiffness reoperation rate of 15.4% and a residual 11° external rotation deficit versus the contralateral shoulder, despite initiating passive ROM on post-operative day 1.^37^ Across the included studies, post-operative immobilization duration ranged from 3 weeks (Porcellini) to 6 weeks (Shields, Voos), with no 2 protocols identical, indicating that no consensus exists in a parameter central to outcomes in both component procedures. A dedicated prospective rehabilitation study in combined repair patients is necessary to further understand the optimal protocol for rehabilitation for this procedure.

Furthermore, this review identifies nerve injury as a morbidity profile that should be studied further. Godinho et al reported an axillary nerve injury rate of 9.1% in a terrible triad presentation. This finding is consistent with the injury mechanism given the nerve's anatomical vulnerability in high-energy anterior dislocation, as prospective consistent with electromyographic studies have identified axillary nerve involvement in up to 42% of acute anterior dislocations.^9^^,^^43^ This suggests that a clinical detection rate of 9.1% likely represents a substantial underestimate of the true neurologic injury burden in this high-energy population. Furthermore, Voos et al^44^ reported persistent abduction weakness in 6 of 16 patients (37.5%), which substantially exceeded the structural retear rate in that cohort (12.5%).^44^ This indicates that functional deficit is not fully explained by mechanical repair failure alone and likely reflects residual neurapraxia or incomplete muscular recovery. Thus, future studies and follow-up protocols should include pre-operative neurologic assessment and post-operative strength testing.

This review has several limitations. The methodological quality of included studies was limited overall, as all 6 studies were retrospective (Level of Evidence III–IV), with none incorporating prospective enrollment, randomization, or blinded outcome assessment. Accordingly, these findings should be interpreted with caution given the limited sample size and low event rates across included studies and the resulting wide CIs should be considered when interpreting the clinical significance of these estimates. Larger, prospective studies are required to validate these observations. However, given the rarity of the injury, prospective randomized study within a single institution is unlikely to be feasible. Due to substantial heterogeneity in PROM instruments included in the studies, quantitative pooling of functional outcomes was not feasible. This heterogeneity may also reflect reporting bias, where studies with more favorable outcomes are more likely to report granular PROM data. Finally, with only 4 to 6 studies contributing to each meta-analyzed outcome, formal assessment of publication bias via Egger test and trim-and-fill analysis is likely underpowered, and these results should be considered supportive rather than definitive. Furthermore, moderator variables such as glenoid bone loss quantification, Goutallier fatty infiltration grade, and pre-operative PROM baselines were absent from all included studies, limiting the ability to identify patients most and least likely to benefit from combined repair. Chan et al,^6^ the largest single cohort in this review, did not report structural retear outcomes, meaning that the primary complication analysis is derived from 5 smaller studies (N = 124) and may not fully represent the demographic profile of the pooled cohort. Future research should prioritize prospective multicenter registry studies with stratification by instability chronicity, standardized computed tomography–based bone loss quantification, and routine post-operative structural imaging and strength assessment to better define the role of combined arthroscopic repair and identify the patients most likely to benefit.

## Conclusion

Single-stage combined arthroscopic repair provides durable anterior stability, with a recurrent instability rate of only 3.3%, but is associated with variable functional outcomes and a structural retear rate of 12.0% that likely exceeds current estimates given inconsistent imaging surveillance across studies. Patient chronicity appears to have a possible association with functional success as acute traumatic presentations in older patients represent the most favorable indication, while chronic recurrent instability with multitendon involvement warrants careful consideration of staged or alternative surgical strategies. Neurologic and functional morbidity beyond redislocation and retear are under-reported in this literature and should be incorporated into standard outcome assessment. High-quality prospective data stratified by chronicity and injury morphology is needed before the role of combined arthroscopic repair can be definitively defined.

## Acknowledgments

The authors would like to thank the University of California, Los Angeles (UCLA) Department of Orthopaedic Surgery for their support during the preparation of this systematic review. All authors had final review and complete control over the publication and writing of this manuscript.

## Disclaimers:

Funding: No funding was disclosed by the authors.

Conflicts of interest: Thomas J Kremen serves as a board or committee member for the AAOS and the American Orthopedic Society for Sports Medicine and is a member of the editorial or governing board for the American Journal of Sports Medicine. Any additional authors, their immediate families, and any research foundations with which they are affiliated have not received any financial payments or other benefits from any commercial entity related to the subject of this article.

## Data availability

All data analyzed during this study are from previously published clinical trials and are included within this article and its supplementary information files. The primary literature sources utilized for this review are publicly available via the PubMed/MEDLINE and Embase databases. Any additional queries regarding the statistical code or data extraction templates used in this meta-analysis may be directed to the corresponding author upon reasonable request.

## Figures and Tables

**Supplementary Figure S1:**
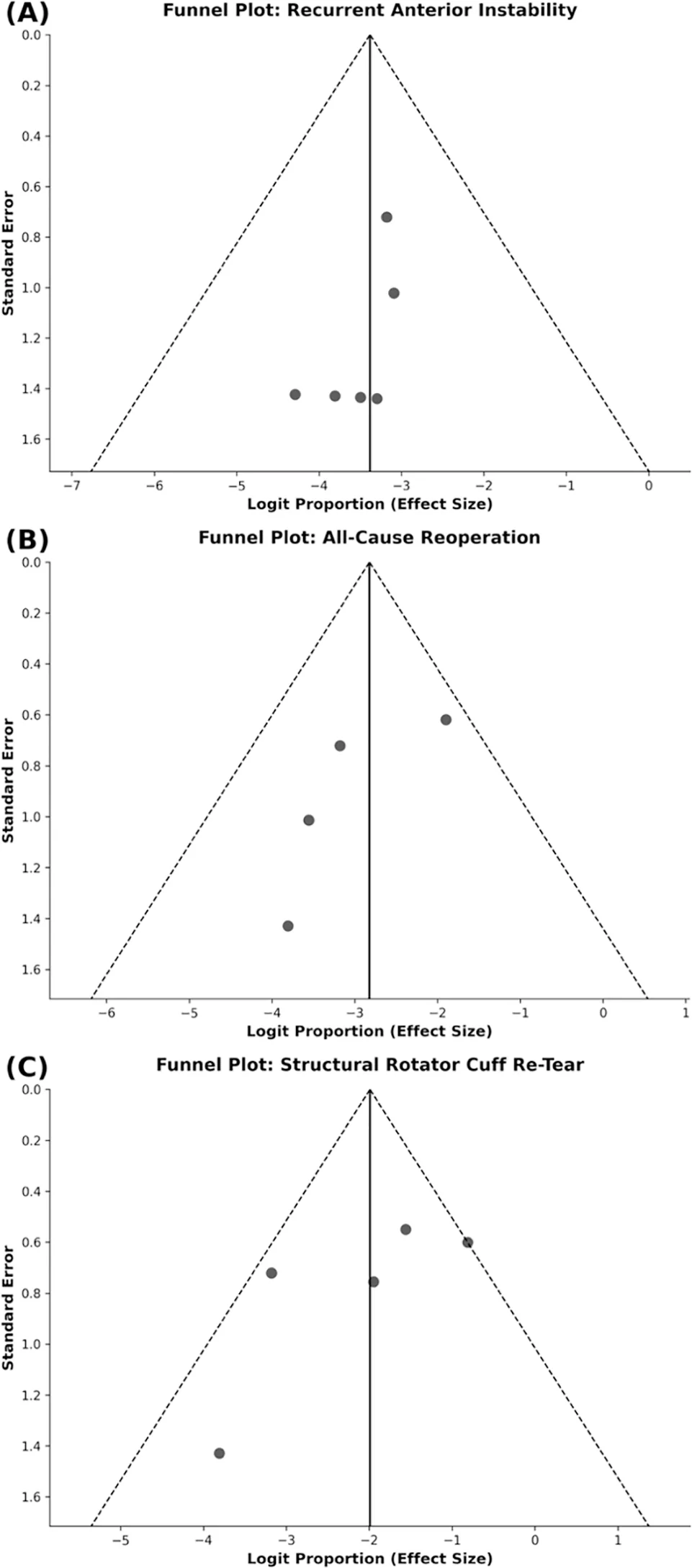


## Supplementary Data

Supplemental

PRISMA checklist
