# Supplementary material for: Clinical outcomes following concomitant arthroscopic rotator cuff repair and anterior shoulder stabilization: a systematic review and meta-analysis
Source: JSES Rev Rep Tech. 2026 Aug 28;6(4):100868. doi: 10.1016/j.xrrt.2026.100868 (PMC13636065; doi:10.1016/j.xrrt.2026.100868)
Supplement: Supplementary file 1 [file mmc1.docx]

**Appendix 1. Full Electronic Search Strategy (PRISMA-S Compliant)**

**Search Overview**

A comprehensive literature search was conducted on **April 6, 2026**, across the following electronic databases. All databases were searched from inception to the date of the search.

**Restrictions:** None (No limits applied for publication date, language, or study design during the initial search).

**Methodology:** Searches used a combination of controlled vocabulary (MeSH in PubMed; Emtree in Embase) and free-text terms.

**Conceptual Framework:** The search logic was built upon four core concepts:

1. Rotator cuff pathology
2. Shoulder instability
3. Combined/concomitant pathology
4. Surgical management (repair/stabilization)

**Search Parameters**

**1. PubMed (MEDLINE)**

**Date Searched:** April 6, 2026

**Results:** 747

**Search String:**

(("Rotator Cuff"[Mesh] OR "Rotator Cuff"[tiab] OR "Rotator Cuff Tear*"[tiab] OR "Supraspinatus"[tiab] OR "Subscapularis"[tiab]) AND ("Shoulder Dislocation"[Mesh] OR "Anterior Shoulder Instability"[tiab] OR "Shoulder Dislocation"[tiab] OR "Bankart Lesion"[tiab] OR "Labral Tear*"[tiab] OR "Glenohumeral Instability"[tiab]) AND ("Combined"[tiab] OR "Concomitant"[tiab] OR "Simultaneous"[tiab] OR "Single-stage"[tiab] OR "One-stage"[tiab]) AND ("Arthroscopy"[Mesh] OR "Arthroscopic"[tiab] OR "Repair"[tiab] OR "Stabilization"[tiab]))

**Notes:**

- MeSH terms were exploded using default PubMed behavior.
- The abbreviation “RCR” was intentionally excluded to minimize retrieval of non-specific results (e.g., *Relative Centrifugal Force*) and improve reproducibility.

**2. Embase (Elsevier)**

**Date Searched:** April 6, 2026

**Results:** 415

**Search String:**

('rotator cuff'/exp OR 'rotator cuff':ti,ab OR 'rotator cuff tear*':ti,ab OR 'supraspinatus':ti,ab OR 'subscapularis':ti,ab) AND ('shoulder dislocation'/exp OR 'anterior shoulder instability':ti,ab OR 'shoulder dislocation':ti,ab OR 'bankart lesion':ti,ab OR 'labral tear*':ti,ab OR 'glenohumeral instability':ti,ab) AND ('combined':ti,ab OR 'concomitant':ti,ab OR 'simultaneous':ti,ab OR 'single-stage':ti,ab OR 'one-stage':ti,ab) AND ('arthroscopy'/exp OR 'arthroscopic':ti,ab OR 'repair':ti,ab OR 'stabilization':ti,ab)

**Notes:**

- Emtree terms were exploded using the **/exp** function.
- No study design or publication-type filters were applied.

**3. Cochrane Central Register of Controlled Trials (CENTRAL)**

**Date Searched:** April 6, 2026

**Results:** 10

**Search String:**

(("rotator cuff" OR supraspinatus OR subscapularis):ti,ab,kw AND ("shoulder instability" OR "shoulder dislocation" OR "bankart lesion" OR "labral tear" OR "glenohumeral instability"):ti,ab,kw AND ("combined" OR "concomitant" OR "simultaneous" OR "single-stage" OR "one-stage"):ti,ab,kw AND ("repair" OR "stabilization" OR "arthroscopic" OR "arthroscopy"):ti,ab,kw)

**Notes:**

- Title, abstract, and keyword fields were searched.
- Strategy aligned conceptually with PubMed and Embase to ensure consistency.

**4. Additional Search Procedures**

- **Deduplication:** Following database export, duplicates were identified and removed using reference management software (Rayyan).
- **Manual Screening:** The reference lists of all included studies and relevant review articles were manually screened to ensure comprehensive capture.
- **Automation:** No automation tools or AI-assisted screening tools were utilized for the search or deduplication process.
