# Supplementary material for: Clinical outcomes following concomitant arthroscopic rotator cuff repair and anterior shoulder stabilization: a systematic review and meta-analysis
Source: JSES Rev Rep Tech. 2026 Aug 28;6(4):100868. doi: 10.1016/j.xrrt.2026.100868 (PMC13636065; doi:10.1016/j.xrrt.2026.100868)
Supplement: Supplementary file 3 [file mmc3.docx]

**Supplemental Figure 1. Publication Bias Funnel Plots**

Funnel plots for each of the three meta-analytic outcomes did not demonstrate clear visual asymmetry, consistent with Egger's regression test results showing no statistically significant publication bias for recurrent instability (p=0.692), structural re-tear (p=0.100), or all-cause reoperation (p=0.215). Trim-and-fill analysis estimated zero missing studies for all three outcomes. Given the small number of studies contributing to each outcome (k=4–6), these analyses are likely underpowered to detect true small-study effects or publication bias, and the absence of statistically significant asymmetry should not be interpreted as definitive evidence against publication bias.
